# Vitamin D Receptor (*VDR*) Gene Polymorphism in Patients Diagnosed with Colorectal Cancer

**DOI:** 10.3390/nu13010200

**Published:** 2021-01-11

**Authors:** Maria Latacz, Dominika Rozmus, Ewa Fiedorowicz, Jadwiga Snarska, Beata Jarmołowska, Natalia Kordulewska, Huub Savelkoul, Anna Cieślińska

**Affiliations:** 1 Faculty of Biology and Biotechnology, University of Warmia and Mazury, 10-719 Olsztyn, Poland; mmlatacz@gmail.com (M.L.); dominika.rozmus@uwm.edu.pl (D.R.); ewa.kuzbida@uwm.edu.pl (E.F.); bj58@wp.pl (B.J.); natalia.smulska@uwm.edu.pl (N.K.); 2 Faculty of Medicine, Collegium Medicum, University of Warmia and Mazury, 10-082 Olsztyn, Poland; 3 Department of General Surgery, Faculty of Medical Sciences, Collegium Medicum, University of Warmia and Mazury, 10-082 Olsztyn, Poland; jadwiga.snarska@uwm.edu.pl; 4 Cell Biology and Immunology Group, Department of Animal Sciences, Wageningen University and Research, 6700 AH Wageningen, The Netherlands; huub.savelkoul@wur.nl

**Keywords:** vitamin D, vitamin D receptor, colorectal cancer, single nucleotide polymorphism (SNP), ApaI, TaqI, FokI, BsmI

## Abstract

Colorectal cancer (CRC) is one of the most commonly occurring neoplasias in humans. The prevalence of CRC rates is still rising. Although the exact background of the disease still remains unknown, it is believed that CRC may not only be a result of environmental factors, but also genetic ones. One of the mechanisms underlying CRC might be the vitamin D pathway, as CRC is the most closely linked neoplasia to vitamin D deficiency. This study shows a possible association of the vitamin D receptor (*VDR*) polymorphisms FokI, BsmI, ApaI, and TaqI with CRC susceptibility. A total of 103 patients diagnosed with CRC (61 men and 42 women, aged 57–82 years) and 109 healthy people (50 men and 59 women, aged 47–68 years) were genotyped using PCR-RFLP for FokI, BsmI, ApaI, and TaqI. None of the single nucleotide polymorphisms (SNPs) individually increased or decreased the risk of CRC. The evaluation of haplotypes revealed two that might enhance the likelihood of CRC development: taB (OR = 30.22; 95% CI 2.81–325.31; *p* = 0.01) and tAb (OR = 3.84; 95% CI 1.29–11.38; *p* = 0.01). In conclusion, genotyping is an easy and robust procedure that needs to be performed only once in a lifetime. A creation of a relevant SNP’s panel might contribute to the identification of the groups that are at the greatest risk of CRC.

## 1. Introduction

According to WHO (World Health Organization) predictions, in 2040 the number of new colorectal cancer (CRC) cases will exceed 3 million and over 1.5 million deaths will be recorded worldwide [1]. Currently, CRC is one of the most commonly occurring neoplasias in humans, as well as one of the most lethal [2]. This is not expected to change in the future, for the Polish population, as well [1].

Less than 5% of CRC cases are cancers in people with genetic predisposition (e.g., familial adenomatous polyposis). Factors increasing the risk of CRC with adequate evidence from scientific reports includes the following: excessive alcohol use, cigarette smoking, obesity, and lack of physical activity [3]. For vitamin D, the data is contradictory, so it is not included on the factor list. However, basic science and preclinical studies strongly suggest that vitamin D is crucial to the appropriate regulation of cancer-relevant pathways: promotion of differentiation and apoptosis with inhibition of proliferation, inflammation, invasion, and metastasis angiogenesis at the same time [4]. Because of its wide availability, low price, and relative safety of supplementation, it is worth avoiding vitamin D deficiency [4].

Moreover, the active form of vitamin D (calcitriol or 1,25(OH)2D) has been demonstrated as inhibiting protumoral action of fibroblasts in CRC stroma via vitamin D receptor (*VDR*) [5]. Its suppressing function of the Wnt/ß-catenin signaling pathway limits proliferation (this pathway is presumably vital to colorectal carcinogenesis) [6,7]. Procancer effect of SNAIL (zinc finger protein SNAI1) might be associated with a reduction of *VDR* expression [8].

**Figure 1:**
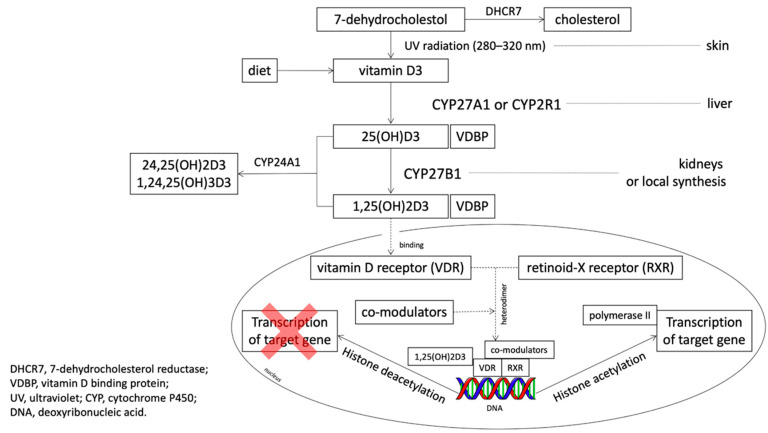
The metabolic pathway of vitamin D (based on Feldman et al., 2014 [4], Jekinson 2019 [13] and Dou et al., 2016 [14] with own modifications).

A steroid intracellular hormone receptor, *VDR* can be liganded only after the addition of two hydroxyl groups (Figure 1). The intestine is recognized as the organ with the highest expression of *VDR* [9]. In one study, *VDR*-null mice were more likely to develop carcinogen-induced cancer [10].

In the case of high *VDR* expression in tumor stromal fibroblasts, overall survival (OS) was better [8]. Apart from *VDR* expression, enzyme *CYP27B1* is also produced, so the cancer cell is capable of generating ligands for *VDR* [11].

**Figure 2:**
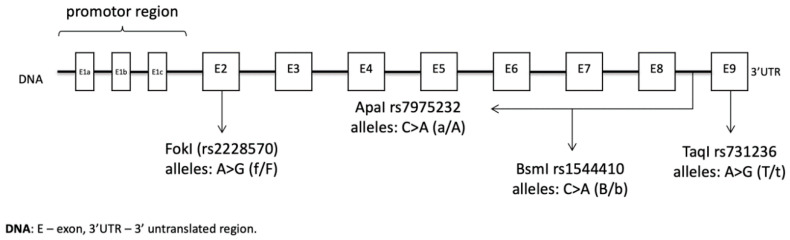
The location and characteristic of *VDR* polymorphism (based on Single Nucleotide Polymorphism Database—dbSNP).

The location of the *VDR* gene is the long arm of chromosome 12 (12q13.11), and it consists of 12 exons [12]. Among numerous single nucleotide polymorphisms (SNPs), a few are most commonly studied: FokI (rs2228570), ApaI (rs7975232), BsmI (rs1544410), and TaqI (rs731236) (Figure 2).

Despite the large amount of research and data collected on polymorphisms in *VDR*, it has not yet been clearly established if any of them may affect the risk of CRC [15]. Therefore, further work is essential. Here, the genetic association of four different *VDR* polymorphisms (ApaI, BsmI, TaqI, and FokI) with susceptibility to colorectal neoplasm was investigated.

## 2. Materials and Methods

### 2.1. Control and Patient Characteristics

Table 1 provides detailed information (sex, age) about our study groups, all of whom were Caucasian. A total of 103 patients diagnosed with CRC (61 men and 42 women, aged 57–82 years, with mean age 71.4 ± 1.8 years) and 109 healthy people (50 men and 59 women, aged 47–68 years, with mean age 58.6 ± 1.3 years) were recruited between 2011 and 2016 at the Independent Public Health Care Institution of the Ministry of the Interior and Administration with the Warmian-Masurian Oncology Center in Olsztyn.

Adenocarcinoma was the diagnosis for all 103 cancer patients, and CRC advancement stages were defined by post-surgical histopathology and clinical evaluation in accordance with TNM Classification of Malignant Tumors. For 23 patients, their tumor stage was I; for 30 patients, their tumor stage was II; for 27 patients, their tumor stage was III; and for 23 patients, their tumor stage was IV. In both cohorts, inflammatory diseases, urogenital tract or other infections, and kidney failures were not observed. This was confirmed by laboratory tests. All required data was collected from patient medical records and/or a filled-in questionnaire. All participants gave informed consent to the study, which was in accordance to ethical standards of the Declaration of Helsinki. It was certified by the Local Bioethics Commission (OIL.492/12/Bioet; 48/2019).

### 2.2. Material Analysis

Approximately 2.0 mL of peripheral blood was collected from 212 participants (103 patients with colorectal cancer and 109 participants in the control group) into a tube containing EDTA (ethylenediaminetetraacetic acid).

GeneJET™ Whole Blood Genomic DNA Purification Mini Kit (Thermo Scientific, Waltham, MA, USA) was used to isolate genomic DNA from peripheral blood nuclear cells; the procedure was performed according to the manufacturer’s protocol.

The chosen method for genotyping all four of the *VDR* polymorphisms (FokI, BsmI, ApaI, and TaqI) was polymerase chain reaction-restriction fragment length polymorphism (PCR-RFLP). The sequences of the primers used for amplification are presented in Table 2 (F—forward primer, R—reverse primer).

PCR amplification was conducted in a thermal cycler according to the following program: initial denaturation: 95 °C for 5 min; proper denaturation: 95 °C for 30 s; attaching the starters at 61 °C for 30 s; synthesis: 72 °C for 60 s; final synthesis: 72 °C for 10 min; number of cycles: 35; cooling: 4 °C.

The mixture for amplification in the volume of 17.5 μL consisted of DreamTaq™ Green Master Mix (Thermo Scientific, Waltham, MA, USA), specific primers, the DNA matrix, and nuclease-free water (Thermo Scientific, Waltham, MA, USA). The yield and specificity of PCR products were evaluated by electrophoresis in 2.5% agarose gel (Promega, Fitchburg, WI, USA) and staining with GelGreen Nucleic Acid Gel Stain (Biotium, Fremont, CA, USA). Amplified fragments were digested with corresponding restriction enzymes shown in Table 2 (Thermo Scientific, Waltham, MA, USA) according to the manufacturer’s instruction and visualized on a 2.5% agarose gel (Table 3). DNA sequencing of random chosen samples after amplification was performed to confirm proper genotyping.

### 2.3. Statistical Analysis

Using chi-square test, the genotype distribution among subjects in the control group was analyzed for Hardy–Weinberg equilibrium (HWE) for each SNP; with the same test, the *p*-value for age and gender was established; standard errors of the mean (SEMs) were counted for age. With SHEsis software, the analysis of linkage disequilibrium (LD), haplotype construction, and general genetic association were performed [19,20]. Odds ratios (ORs) and 95% confidence intervals (CIs) were calculated to set the association between alleles and genotypes. Statistical analysis was conducted on IBM SPSS Statistics 26 (IBM, Armonk, NY, USA) and GenAlEx 6.502 [21] software, with ≤0.05 *p*-value considered statistically significant, excluding logistic regression: ≤0.0125 adjusted *p*-value (according to Bonferroni correction).

## 3. Results

The distribution of all four SNPs genotypes were in Hardy–Weinberg equilibrium in the control group (Table 4).

Table 1 presents the clinical characteristics of the study participants. A discrepancy can be noted between the percentage of men and women in the study and control cohorts When it comes to tumor staging, stage II was the most frequently encountered in histopathology examination. Stage II is localized or node-negative disease, while stage III is regional or node-positive disease [22]. The tumor itself was classified as T3, meaning that the tumor grew through muscularis propria and into subserosa (it is not a total occupation of colorectal wall) or the cancerous cells appeared in the surrounding tissue [22]. The majority of CRC patients had metastasis to lymph nodes but not to distant organs.

Table 5 presents the distribution of alleles and genotypes at the polymorphic sites as well as associations between the genotype and CRC incidence. For TaqI polymorphism, allele t was far more common in the CRC group (43%) than in the control group (29%). At first, a comparison of genotypes TT vs. Tt, then TT vs. tt showed that TT decreased the risk of CRC by over two times (OR = 0.39; 95% CI 0.22–0.72; *p* = 0.002 and OR = 0.37; 95% CI 0.15–0.89; *p* = 0.027, respectively). Possessing at least one allele t at the polymorphic site seemed to be a CRC risk factor (Tt + tt vs. TT: OR = 2.60; 95% CI 1.66–4.07; *p* < 0.0001, T vs. t: OR = 0.55; 95% CI 0.37–0.82; *p* = 0.003). However, after performing logistic regression, neither TaqI genotypes nor alleles showed a statistically significant association with CRC susceptibility (for *p* ≤ 0.0125; TT vs. Tt: adjusted OR = 0.36; 95% CI 0.15–0.83; *p* = 0.016, TT vs. tt: adjusted OR = 0.31; 95% CI 0.09–1.09; *p* = 0.68, T vs. t: adjusted OR = 0.50; 95% CI 0.29–0.88; *p* = 0.017).

No correlation between ApaI genotypes or alleles and susceptibility of CRC was observed. The same was observed for FokI.

In the case of the BsmI polymorphism, allele b looked to be protective against CRC (bb vs. BB + Bb: OR = 1.64; 95% CI 1.05–2.54; *p* = 0.0287). However, logistic regression did not confirm those results (bb vs. Bb: OR = 1.05; 95% CI 0.30–3.71; *p* = 0.19; bb vs. BB: adjusted OR = 1.73; 95% CI 0.77–3.91; *p* = 0.93; b vs. B: adjusted OR = 1.20; 95% CI 0.69–2.11; *p* = 0.52).

**Figure 3:**
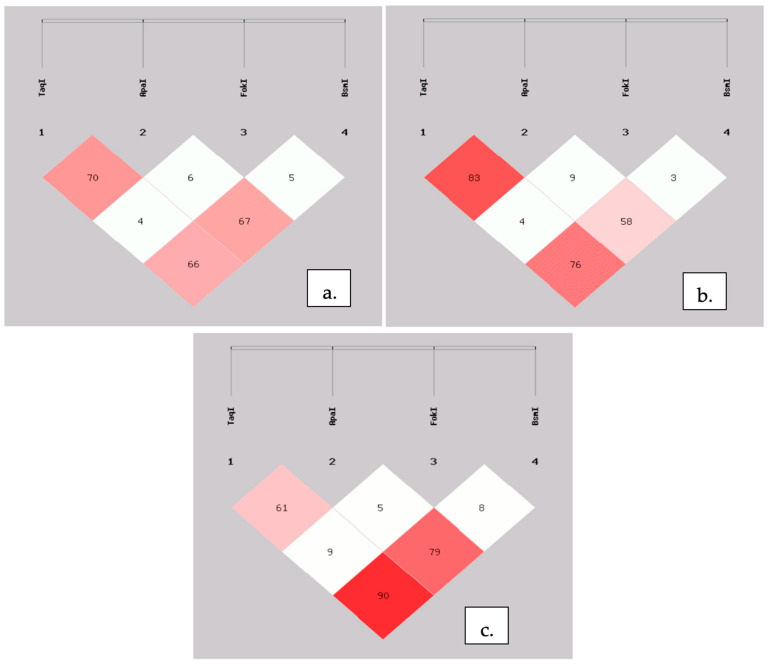
Linkage disequilibrium plots for four investigated SNPs for both groups (**a**), controls (**b**), and CRC patients (**c**). The number represents the percentage value of ‘D, which is also indicated by color intensity.

With SHEsis software, we created haplotypes plots (Figure 3). Obtained results were quite similar to those presented in LDlink tool for the European population [23]. For further analysis of haplotypes, FokI polymorphism was excluded, on account of its very low LD between other SNPs (Figure 3).

We identified two haplotypes that significantly increase the risk of CRC. For tAb nearly a fourfold increase in risk was observed (OR = 3.84; 95% CI 1.29–11.38; *p* = 0.01), while for taB the likelihood of malignancy in colon or rectum was noted to be thirty times greater (OR = 30.22; 95% CI 2.81–325.31; *p* = 0.01) (Table 6).

Table 7 presents the frequencies of BsmI, ApaI, and TaqI combined genotypes in the CRC group and in the control group. Comparing the genotypes with ttAaBb, ttaaBB, or ttAAbb as the reference genotype (possession of two “risky” haplotypes) could be highly questionable, since their prevalence is very low. Instead, TtAaBb was chosen because it possessed one risk-increasing haplotype and was relatively frequent in the study groups. It was found more often in the control group (30.28%). Our further analysis included only combined genotypes with a frequency of at least 3% in one of the groups. The results were as follows: TtAaBb vs. TTaaBb: OR = 19.78; 95% CI 1.12–349.60; *p* = 0.04; TtAaBb vs. TtAabb: OR = 0.07; 95% CI 0.01–0.59; *p* = 0.01 and, interestingly, TtAaBb vs. Ttaabb: OR = 0.10; 95% CI 0.01–0.84; *p* = 0.03.

## 4. Discussion

Due to the current and projected increasing number of CRC cases and associated mortality, this neoplasm is expected be one of the greatest burdens in oncology. Determining further factors influencing the etiopathogenesis or the course of the illness should be a priority.

It was previously mentioned that less than 5% of CRC cases are cancers in people with genetic predisposition [3], but heritable factors explain about 35% of the risk of CRC [24] and may influence the variability of vitamin D status [25]. The location of three out of four polymorphisms (ApaI, TaqI, and BsmI) is 3′UTR, and this region is mainly responsible for expression regulation (mRNA stability) [26]. Therefore, it is possible for these polymorphisms to disturb vitamin D pathway.

The obtained results (Table 5) first suggested a significant impact of TaqI and BsmI polymorphisms on the occurrence of CRC. Collected data indicated a potential unprotective role of the t allele (Tt + tt vs. TT: OR = 2.60; 95% CI 1.66–4.07; *p* < 0.0001; T vs. t: OR = 0.55; 95% CI 0.37–0.82; *p* = 0.003), but after performing logistic regression the genotypes did not show any correlations (TT vs. Tt: adjusted OR = 0.36; 95% CI 0.15–0.83; *p* = 0.016; TT vs. tt: adjusted OR = 0.31; 95% CI 0.09–1.09; *p* = 0.068). The *p*-value for TT vs. Tt was nearly statistically significant, but it slightly exceeded 0.0125. There was a similar situation for BsmI: initially, it was flagged as potentially being a CRC prognostic factor, but not after adjustment (bb vs. Bb: adjusted OR = 1.05; 95% CI 0.30–3.71; *p* = 0.19, bb vs. BB: adjusted OR = 1.73; 95% CI 0.77–3.91; *p* = 0.93).

A Polish population-based study with a similar number of participants demonstrated TT genotype as a risk factor and also a statistically significant association for ApaI [27]. Touvier et al. created a meta-analysis that included 23 articles reflecting on *VDR* polymorphisms in colorectal cancer. They found only the BsmI polymorphism to be statistically significant in lowering neoplasm risk (RR = 0.57, 95% CI: 0.36–0.89 for BB vs. bb) [28]. Another meta-analysis included 10 articles, and the authors established [29] an association between CRC risk and TaqI polymorphism (OR = 1.43, 95% CI: 1.30–1.58 for tt vs. TT).

A meta-analysis based on 39 case-control studies showed not only polymorphisms Fok I, Bsm I, Apa I, and Taq I but also Cdx2 as being possible risk factors for CRC. However, the study did not show a significant correlation between Apa I, Taq I, Bsm I, Fok I, Cdx2, and higher CRC risk [30]. On the other hand, a study confirmed the existing association of vitamin D deficiency with increased risk of CRC and mortality. All of the four *VDR* polymorphisms were revealed as significant among cancer individuals with the following homozygous mutant genotypes: aa, bb, ff, and tt. Patients with stage IV CRC had all of those mutant genotypes. The result suggests there may be higher mortality among patients with homozygous mutant genotypes compared with heterozygous ones [31].

Investigations of haplotypes and combined genotypes is not present in as many papers as sole polymorphism impacts on CRC risk. Furthermore, it can be difficult to compare the results between works establishing associations for different polymorphisms at the same time because of different blocks of SNPs.

For our study, two haplotypes that put the carrier at greater risk were identified: tAb (OR = 3.84; 95% CI 1.29–11.38; *p* = 0.01) and especially taB (OR = 30.22; 95% CI 2.81–325.31; *p* = 0.01).

For example, in a previous study on the Thai population, none of the polymorphisms modulated the risk of CRC singly. However, AGGT was found to be protective [32]. Translating this into our manner of showing haplotypes, this would T, a, b at the polymorphic sites, with an allele at Tru9I that was not taken into account in our study. These findings are similar to ours; for single polymorphisms, the results are mixed or not statistically significant, but there are valuable findings after the haplotypes analysis presents the most important haplotypes. A study based on the Newfoundland population investigated TaqI and BsmI polymorphism in one block for CRC survival and found no relevant association [33]. Ashmore et al. determined null results with CRC for both sole SNPs in *VDR* gene and haplotypes [34].

Apart from considering the direct correlation between CRC occurrence and SNPs, there are studies that reflect on the association in an indirect way. Al-Ghafari et al. investigated a possible relationship between serum 25(OH)D and polymorphism. Their findings indicated that ApaI is an important factor affecting the level of vitamin D and that TaqI is a modificatory of serum calcium level [35].

Higher intakes of vitamin D and calcium have been associated with almost a 50% reduction in colorectal cancer risk among the Chinese population [36].

We are fully aware of the limitations in our study in that our study groups were limited in size and our study participants represented only one race and one nationality. That could be the reason for contradictory results for haplotypes and combined genotypes—a genotype with an earlier identified risky haplotype decreased the risk of CRC compared to a genotype without such an allele (TtAaBb vs. Ttaabb: OR = 0.10; 95% CI 0.01–0.84; *p* = 0.03). Nevertheless, in two other cases, the calculated value of OR was the same as expected due to the presence of a risky haplotype (TtAaBb vs. TTaaBb: OR = 19.78; 95% CI 1.12–349.60; *p* = 0.04; TtAaBb vs. TtAabb: OR = 0.07; 95% CI 0.01–0.59; *p* = 0.01).

Our conclusion is that genotyping is an easy and robust procedure that needs to be performed only once in a lifetime. The creation of a relevant SNPs panel/haplotypes might contribute to the identification of people at the greatest risk of CRC and help direct them toward receiving dedicated oncologic surveillance. The investigation of haplotypes in CRC is not as well represented in science as separate investigations of SNPs. Our study contributed to the identification of *VDR* haplotypes in representatives of a European population with one of the highest mortalities (current: 59% and expected in 20 years: 64%) [1].

## Figures and Tables

**Table 1:** Clinical characteristics of patients and participants in the control group. Numbers in brackets indicate the percentage of patients relative to the total group.

	CRC Group (n = 103)	Control Group (n = 109)	*p*-Value
**Age: years (mean ± SEM)**	71.4 ± 0.8	58.6 ± 0.6	<0.01
**Gender, n (%)**			
Female	42 (41)	59 (54)	0.052
Male	61 (59)	50 (46)	
**Tumor stage, n (%)**			
I	23 (22.3)		
II	30 (29.1)		
III	27 (26.3)		
IV	23 (22.3)		
**Pathological tumor stage (pT), n (%)**			
T1	0 (0)		
T2	21 (20.4)		
T3	63 (61.2)		
T4	19 (18.4)		
**Pathological nodal status (pN), n (%)**			
no lymph node metastasis	49 (47.6)		
lymph node metastasis	54 (52.4)		
**Pathological metastasis status (pM), n (%)**			
no distant metastasis	64 (62.1)		
metastasis to distant organs	39 (37.9)		

**Table 2:** Primers for single nucleotide polymorphisms (SNPs) in the *VDR* gene and restriction enzymes.

SNP	Primer Sequence	Restriction Enzyme	Source
FokI	Fok1R: 5-ATGGAAACACCTTGCTTCTTCTCCCTC-3 Fok11F: 5-AGCTGGCCCTGGCACTGACTCtGGCTCT-3	FastDigest Fok-I	Maalmi et al., 2013, Mansour et al., 2010 [16,17] with own modifications
ApaI	ATaq1F: 5-GGATCCTAAATGCACGGAGA-3 ATaq1R: 5-AGGAAAGGGGTTAGGTTGGA-3	FastDigest Apa-I	Designed with Primer 3 application [18]
BsmI	ABsm1F: 5-CGGGGAGTATGAAGGACAAA-3 ABsm1R: 5-CCATCTCTCAGGCTCCAAAG-3	FastDigest Hha-I	Designed with Primer 3 application [18]
TaqI	ATaq1F: 5-GGATCCTAAATGCACGGAGA-3 ATaq1R: 5-AGGAAAGGGGTTAGGTTGGA-3	FastDigest Taq-I	Designed with Primer 3 application [18]

**Table 3:** Pattern for genotyping.

SNP	Pattern for Genotyping
FokI	FF homozygote: 267 bp Ff heterozygote: 267, 198, 69 bp ff homozygote: 198, 69 bp PCR product: 267 bp
ApaI	AA homozygote: 630 bp Aa heterozygote: 630, 484, 146 bp aa homozygote: 484, 146 bp PCR product: 630 bp
BsmI	BB homozygote: 348 bp Bb heterozygote: 348, 243, 105 bp bb homozygote: 243, 105 bp PCR product: 348 bp
TaqI	TT homozygote: 425, 205 bp Tt heterozygote: 425, 225, 205, 200 bp tt homozygote: 225, 205, 200 bp PCR product: 630 bp

**Table 4:** Hardy–Weinberg equilibrium for the control group.

SNP	x^2^	*p*-Value
FokI	0.563	0.755
ApaI	0.120	0.942
BsmI	3.218	0.200
TaqI	0.580	0.748

**Table 5:** Genotype and allele frequencies of *VDR* polymorphisms in CRC and control groups.

Polymorphism	Genotype/ Allele	CRC n (%)	Control n (%)	Crude OR (95% CI)	*p*-Value	Adjusted OR (95% CI)	*p*-Value
	TT	30 (29)	56 (51)	-	-	-	-
TaqI	Tt	57 (55)	42 (39)	0.39 (0.22–0.72)	0.002	0.36 (0.15–0.83)	0.016
rs731236	tt	16 (16)	11 (10)	0.37 (0.15–0.89)	0.027	0.31 (0.09–1.09)	0.068
	T	117 (57)	154 (71)	-	-	-	-
	t	89 (43)	64 (29)	0.55 (0.37–0.82)	0.003	0.50 (0.29–0.88)	0.017
Tt + tt		89 (60)	64 (36)	-	-	-	-
vs. TT		60 (40)	112 (64)	2.60 (1.66–4.07)	<0.0001	-	-
	AA	23 (22)	19 (17)	-	-	-	-
ApaI	Aa	49 (48)	55 (50)	1.36 (0.63–2.79)	0.40	0.81 (0.29–2.25)	0.68
rs7975232	aa	31 (30)	35 (32)	1.37 (0.63–2.97)	0.43	0.81 (0.40–3.72)	0.72
	A	95 (46)	93 (43)	-	-	-	-
	a	111 (54)	125 (57)	1.13 (0.78–1.67)	0.51	1.15 (0.67–1.98)	0.60
Aa + aa		111 (71)	125 (77)	-	-	-	-
vs. AA		46 (29)	38 (23)	1.36 (0.83–2.25)	0.22	-	-
	FF	32 (31)	43 (39)	-	-	-	-
FokI	Ff	50 (49)	48 (44)	0.71 (0.39–1.31)	0.28	0.70 (0.29–1.65)	0.41
rs2228570	ff	21 (20)	18 (17)	0.64 (0.29–1.39)	0.26	0.58 (0.19–1.72)	0.32
	F	114 (55)	134 (61)	-	-	-	-
	f	92 (45)	84 (39)	0.78 (0.53–1.14)	0.20	0.74 (0.43–1.28)	0.28
Ff + ff		92 (59)	84 (48)	-	-	-	-
vs. FF		64 (41)	86 (52)	1.47 (0.95–2.28)	0.08	-	-
	bb	50 (49)	38 (35)	-	-	-	-
BsmI	Bb	40 (39)	60 (55)	1.97 (1.10–3.53)	0.02	1.05 (0.30–3.71)	0.19
rs1544410	BB	13 (13)	11 (10)	1.11 (0.45–2.76)	0.82	1.73 (0.77–3.91)	0.93
	b	140 (68)	136 (62)	-	-	-	-
	B	66 (32)	82 (38)	0.78 (0.52–1.17)	0.23	1.20 (0.69–2.11)	0.52
bb vs.		66 (40)	82 (52)	-	-	-	-
BB + Bb		100 (60)	76 (48)	1.64 (1.05–2.54)	0.03	-	-

**Table 6:** Haplotypes with alleles in linkage disequilibrium (LD): ApaI, TaqI, and BsmI.

Haplotype	CRC Frequency (%)	Control Frequency (%)	OR (95% CI)	*p*-Value
TAB	1.7	3.8	0.43 (0.12–1.53)	0.18
TAb	10.2	12.1	2.84 (0.46–1.53)	0.56
TaB	0.0	8.9	-	-
Tab	44.9	45.9	0.96 (0.65–1.40)	0.82
tAB	27.0	24.8	1.12 (0.72–1.73)	0.61
tAb	7.2	2.0	3.84 (1.29–11.38)	0.01
taB	3.4	0.1	30.22 (2.81–325.31)	0.01
tab	5.6	2.4	2.39 (0.84–6.82)	0.09

**Table 7:** Combined genotypes and their percentage among investigated populations.

Genotype	CRC n (%)	Control n (%)	*p*-Value
TtAaBb	26 (25.24)	33 (30.28)	-
TtAabb	11 (10.68)	1 (0.92)	0.01
TTAaBb	1 (0.97)	8 (7.34)	0.92
TTAabb	8 (7.77)	11 (10.09)	0.88
ttAABB	10 (9.71)	8 (7.34)	0.39
TTaaBb	0	12 (11.01)	0.04
TTaabb	19 (18.45)	19 (17.43)	0.56
TtAABb	6 (5.83)	3 (2.75)	0.22
Ttaabb	8 (7.77)	1 (0.92)	0.03
TtAAbb	2 (1.94)	1 (0.92)	-
TTAABb	1 (0.97)	2 (1.83)	-
TTAAbb	1 (0.97)	3 (2.75)	-
TTaaBB	0	1 (0.92)	-
TtAaBB	0	1 (0.92)	-
TtaaBb	3 (2.91)	1 (0.92)	-
TtAABB	1 (0.97)	1 (0.92)	-
ttAAbb	1 (0.97)	1 (0.92)	-
ttAABb	1 (0.97)	0	-
ttAaBb	2 (1.94)	1 (0.92)	-
ttAaBB	1 (0.97)	0	-
ttaaBB	1 (0.97)	0	-
ttaabb	0	1 (0.92)	-

## Data Availability

Detailed information is available upon request.
